# Fixed-Life or Rechargeable Batteries for Deep Brain Stimulation: Preference and Satisfaction Among Patients With Hyperkinetic Movement Disorders

**DOI:** 10.3389/fneur.2021.662383

**Published:** 2021-05-28

**Authors:** Xian Qiu, Yuhan Wang, Zhengyu Lin, Yunhao Wu, Wenying Xu, Yiwen Wu, Bomin Sun, Keyoumars Ashkan, Chencheng Zhang, Dianyou Li

**Affiliations:** ^1^Department of Nursing, Ruijin Hospital, Shanghai Jiao Tong University School of Medicine, Shanghai, China; ^2^Department of Neurosurgery, Ruijin Hospital, Shanghai Jiao Tong University School of Medicine, Shanghai, China; ^3^Department of Neurology, Ruijin Hospital, Shanghai Jiao Tong University School of Medicine, Shanghai, China; ^4^Department of Neurosurgery, King's College Hospital, London, United Kingdom

**Keywords:** movement disorders, implantable pulse generators, deep brain stimulation, hyperkinetic movement disorders, dystonia, Tourette syndrome

## Abstract

**Background:** Deep brain stimulation (DBS) is an established treatment for hyperkinetic movement disorders. Patients undergoing DBS can choose between the use of a rechargeable or non-rechargeable battery for implanted pulse generators (IPG).

**Objectives:** In this study, we aimed to evaluate patient preferences and satisfaction with rechargeable and non-rechargeable batteries for IPGs after undergoing DBS.

**Methods:** Overall, 100 patients with hyperkinetic movement disorders (dystonia: 79, Tourette syndrome: 21) who had undergone DBS took a self-designed questionnaire to assess their satisfaction and experience with the type of battery they had chosen and the factors influencing their choice.

**Results:** Of the participants, 87% were satisfied with the stimulating effects of the treatment as well as the implanted device; 76% had chosen rechargeable devices (r-IPGs), 71.4% of whom recharged the battery themselves. Economic factors were the main reason for choosing both r-IPG and non-rechargeable IPG (nr-IPG). The questionnaire revealed that 66% of the patients checked their r-IPG battery every week. The mean interval for battery recharge was 4.3 days.

**Conclusions:** The majority of the patients were satisfied with their in-service-IPG, regardless of whether it was a r-IPG or nr-IPG. Affordability was the main factor influencing the choice of IPG. The majority of the patients were confident in recharging the battery of their r-IPG themselves; only 11% of patients experienced difficulties. Understanding the recharge process remains difficult for some patients and increasing the number of training sessions for the device may be helpful.

## Introduction

Deep brain stimulation (DBS) has been widely used in the treatment of several movement disorders, including Parkinson's disease (PD) (1), and hyperkinetic movement disorders, including dystonia (2) and Tourette syndrome (3). DBS treatment for hyperkinetic movement disorders differs from that for PD in the stimulation targets, stimulation parameters, and battery consumption. It has also been reported that dystonia patients tend to be less satisfied with rechargeable DBS devices than patients with PD (4). Thus, we evaluated patient preference and satisfaction with implanted pulse generators (IPGs) separately among those with PD and those with hyperkinetic movement disorders. In this study, we focused on patients with hyperkinetic movement disorders.

IPGs for the treatment of DBS include rechargeable IPGs (r-IPGs) and non-rechargeable IPGs (nr-IPGs). Nr-IPGs were the only option available to patients requiring DBS until r-IPGs were introduced in 2008. Patients with nr-IPGs face an important limitation, namely, IPG depletion, which requires an obligatory surgery for replacement. Compared with a battery life of 3–5 years among patients with PD (5). nr-IPG batteries last only 1–3 years among patients with dystonia (6). Battery consumption is proportional to the intensity of DBS, and patients requiring a higher intensity will require more frequent IPG replacement. It has been reported that the infection rate increases with multiple IPG replacements; for instance, the infection rate reached more than 20% with three and four IPG replacements (7).

IPG replacement leads to not only an increased infection rate, but also a higher long-term cost. The age of onset of PD is ~65–70 years (8), and the mean disease duration until death ranges from 10 to 20 years, with significant heterogeneity (9, 10). Among hyperkinetic movement disorders, the age of disease onset varies: focal limb dystonia manifests in the 30s, cervical dystonia manifests in the 30s and 40s (11), and Tourette syndrome manifests before 18 years of age, mainly from 4 to 12 years (12). Overall, the age of onset in hyperkinetic movement disorders is younger than that in PD, and the mean disease duration until death is longer. This means that DBS is required for a longer period in patients with hyperkinetic movement disorders, and those with nr-IPGs will require more IPG replacements. Although the initial price of r-IPG is higher than that of nr-IPG, the cost of nr-IPG replacements increases over time. Hitti et al. (13) reported a savings of ~60,900 USD for those with r-IPGs when compared to those with nr-IPGs over the course of 9 years, and Rizzi et al. (14) reported the cost of IPG replacement and complication management to be €234,194 during a mean follow-up period of 7.9 years. Although the price of IPG varies in different countries and regions, the r-IPG is more cost-effective in the long-term. Considering the longer DBS usage time in patients with hyperkinetic movement disorders, r-IPG seems to be a better choice for long-term cost savings.

Regarding the experience of using an IPG, patients have shown a preference for r-IPGs in several studies (13, 15, 16), which is similar to the clinical effect. Although r-IPGs do not require battery replacement in a relatively short period of time, they involve the hassle of the recharging process. Patients and their caregivers must learn to use the recharging device, and the IPG should be checked and recharged routinely (often more frequently than once a week). R-IPG affects patients' daily lives, and it is often a challenge to recharge an IPG during work or travel.

To determine the Chinese patients' actual experiences using different kinds of IPGs, we recruited 100 patients with dystonia or Tourette syndrome to participate in a survey. We discuss herein the differences in preference and satisfaction with nr-IPGs and r-IPGs among patients with hyperkinetic movement disorders.

## Materials and Methods

### Patients

Questionnaires were collected from 100 patients with hyperkinetic movement disorders (dystonia: 79, Tourette syndrome: 21) who had undergone DBS surgeries at the Center for Functional Neurosurgery within the Department of Neurosurgery at Ruijin Hospital (affiliated with Shanghai JiaoTong University School of Medicine). Patients implanted with nr-IPGs or r-IPGs were included. The questionnaires inquired about their satisfaction with and preference for DBS devices. As stated at the top of the questionnaire, completion of the survey by participants was considered to imply consent for data collection and analysis. The Ruijin Ethical Committee approved this study, and the study was conducted in accordance with the Declaration of Helsinki.

### Questionnaire

An Internet-based questionnaire (powered by www.wjx.cn) was developed and distributed via the online chat program, WeChat. The questionnaire was designed with reference to the research of Jakobs et al. (16), and were adjusted according to the situation of Chinese patients. The questions covered patient demographics, factors that impacted the patient's choice, the patient's satisfaction with their choice, and DBS surgery. In particular, several questions were designed specifically for patients implanted with an r-IPG device; they inquired about the feasibility and reliability of the battery recharge, the interval between recharges, the duration of the recharge process, and the convenience of postoperative r-IPG management. The questionnaire was distributed via the online chat platform WeChat. Participants completed the questionnaire after having received at least 8 months of DBS treatment. In most cases, it took no more than 30 min to complete the questionnaire.

### Statistical Analyses

SPSS software (version 23.0. Armonk, NY, IBM Corp.) was used for the data analysis. Continuous variables are expressed as the mean ± standard deviation or as the median value with the interquartile range (IQR). Categorical variables are presented as frequencies (%). The Fisher's exact-test was used to assess the association between affordability and choice of IPG. The Pearson's Chi-square-test or Fisher's exact-test was used to evaluate the magnitude of the impact of battery size, the need for battery replacement, the need for battery recharging, and economic issues regarding the patient's choice of r-IPG vs. nr-IPG. We used Yates' correction for continuity to compare the satisfaction rates between patients with r-IPGs and nr-IPGs.

## Results

### Demographic Data

Seventy-nine patients diagnosed with dystonia and 21 with retractable Tourette syndrome completed the survey and were included in the analysis. Among them, 76 were implanted with an r-IPG and 24 were implanted with an nr-IPG. The mean age was 38.1 ± 17.8 years, and the median follow-up duration was 17 (7.25–34) months. The demographic characteristics are shown in Table 1.

**Table 1:** Demographic data.

**Characteristics**	**Total (*N* = 100)**	**r-IPG (*N* = 76)**	**nr-IPG (*N* = 24)**
**Sex**			
Men	60	50	10
Women	40	26	14
**Age, years (Mean** **±** **SD)**	38.1 ± 17.8	37.6 ± 17.7	39.7 ± 18.1
**Follow-up, months (Median, IQR)**	17 (7.25–34)	13 (6.25–28.75)	30 (18.5–57.25)

*SD, standard deviation; IQR, interquartile range; r-IPG, rechargeable implanted pulse generator; nr-IPG, non-rechargeable implanted pulse generator*.

### Factors Influencing Patients' Choice of Device

**Figure 1:**
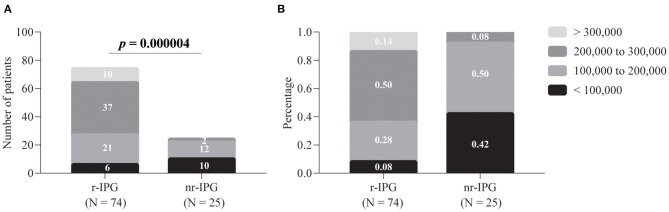
Patient budgets for deep brain stimulation and implanted pulse generators (IPGs). The budgets are divided into four levels. Data are presented as either absolute numbers **(A)** or percentages **(B)**. A *p*-value < 0.05 is considered statistically significant. r-IPG, rechargeable IPG; nr-IPG, non-rechargeable IPG.

**Figure 2:**
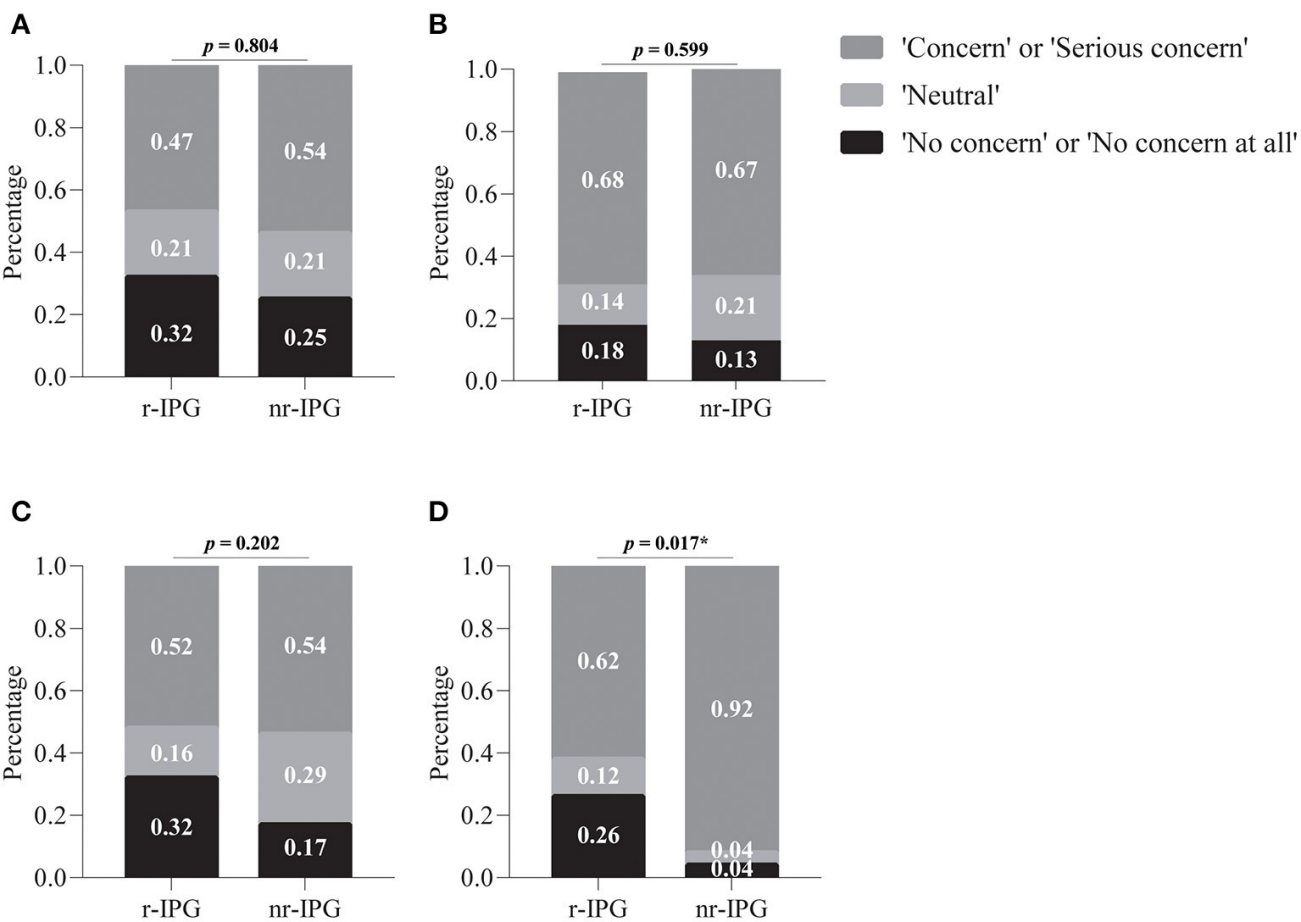
Factors influencing patients' choice between a rechargeable (r-IPG) and non-rechargeable (nr-IPG) implanted pulse generator. **(A)** Battery size; **(B)** the need for further surgery to replace the battery; **(C)** the need for recharging the battery, and **(D)** economic issues. Patients' attitudes toward these factors were divided into five levels in the questionnaire: “No concern at all,” “No concern,” “Neutral,” “Concern,” and “Serious concern.” The choices of “Concern” and “Serious concern,” as well as “No concern” and “No concern at all” were merged for data analysis. Data are presented as percentages. *p-value < 0.05 was considered statistically significant.

As shown in Figure 1, 50% of the patients (37/74) who chose r-IPG had a budget between 200,000 and 300,000 RMB; only 8% of the patients (2/24) who chose nr-IPG could afford the same. The majority of the patients (92%, 22/24) who chose nr-IPG reported a budget below 200,000 RMB. The choice of r-IPG vs. nr-IPG was significantly associated with affordability (*p* = 0.000004). Specifically, the percentage of patients reporting concern regarding economic issues was significantly higher among those who chose nr-IPG (92%, 22/24) than among those who chose r-IPG (62%, 47/76; *p* = 0.017) (Figure 2D). Similar proportions of patients with r-IPG and with nr-IPG reported concern regarding battery size (47%, 36/76 vs. 54%, 13/24, respectively; *p* = 0.918), the need for further surgery to replace the battery (68%, 52/76 vs. 67%, 16/24, respectively; *p* = 0.599), and the need for recharging the battery (52%, 40/76 vs. 52%, 13/24, respectively; *p* = 0.202) (Figures 2A–C).

### Satisfaction

Overall, the majority of the patients (87%, 87/100) were satisfied with the stimulating effects as well as the implanted device. Only nine (9%) claimed that the stimulating effects did not meet their expectations. The satisfaction rate among patients with r-IPGs (86%, 65/76) did not differ significantly from that among patients with nr-IPGs (92%, 22/24; *p* = 0.666) (Table 2). A total of 82% (62/76) of patients with r-IPGs and 92% (22/24) with nr-IPGs would choose the same type of device at the time of the survey.

**Table 2:** Satisfaction rate.

**Questions**	**Group**	
	**r-IPG**	**nr-IPG**
	**(*N* = 76)**	**(*N* = 24)**
**1. Are you still happy with your choice of device?**		
Yes	65 (86%)	22 (92%)
No	11 (14%)	2 (8%)
** 1.1. If not, please specify the reason**.		
The stimulating effects did not meet your expectations.	9 (12%)	0
Other	2 (3%)	2 (8%)
**2. Would you choose the same type of device today?**		
Yes	62 (82%)	22 (92%)
No	14 (18%)	2 (8%)

*r-IPG, rechargeable implanted pulse generator; nr-IPG, non-rechargeable implanted pulse generator*.

### Surgery-Related Complications

Three patients (3%, 3/100) suffered from intracranial infection after DBS implantation. No other complications (e.g., hemorrhage, neurological deficit) were reported after surgery for IPG replacement. No complications occurred after the re-operation (battery replacement).

### Stimulation-Related Complications

Battery depletion occurred in 4 of 24 patients (16.7%) with nr-IPGs. All 4 patients had dystonia and did not discover that the battery had been depleted until their symptoms reappeared and aggravated. Battery depletion also occurred in 1 patient (1.3%, 1/76) with an r-IPG. The patient had Tourette's syndrome and stated that the battery's depletion had led to more severe symptoms than those experienced before the surgery. The symptoms disappeared after recharging of the battery.

### Recharging Process

The majority of the r-IPG patients (82%, 62/76) were capable of checking and recharging the battery independently. The remaining (18%, 14/76), however, required help to check and recharge the battery. Most of the patients with r-IPGs or their caretakers (89%, 68/76) reported feeling confident using the device. Of these, 38% (26/68) reported confidence within 1 week of discharge. Another 36% (24/68) of them required more than 4 weeks to gain confidence. More than half of the patients (66%, 50/76) checked the battery every week, and most (87%, 66/76) preferred recharging when the battery level was over 50%. The mean interval for battery recharge was 4.3 days. Half of the patients (50%, 38/76) spent more than 1 h recharging. A few patients (28%, 21/76) reported forgetting to recharge. Notably, 13% (10/76) of r-IPG patients reported at least one experience of being unable to recharge the battery, and half of these patients could not perform the troubleshooting themselves (50%, 5/10) (Tables 3, 4).

**Table 3:** Recharging process for patients with rechargeable implanted pulse generators (r-IPGs) (*N* = 76) (Question 1–5).

**Questions**	**Number (%)**
**1. Do you feel confident using your r-IPG?**	
No	8 (11%)
Yes	68 (89%)
** 1.1. If yes, how long did it take for you to feel confident?**	
<1 week	26 (38%)
1–2 weeks	13 (19%)
2–4 weeks	5 (7%)
More than 4 weeks	24 (36%)
**2. How frequently do you check the battery capacity of your r-IPG?**	
Every day	13 (17%)
Every week	50 (66%)
Every 2 weeks	5 (7%)
Every 4 weeks	4 (5%)
Every year	4 (5%)
**3. Do you ever forget to recharge your r-IPG?**	
No	55 (72%)
Yes	21 (28%)
**4. How frequently do you recharge your r-IPG?**	
Every day	16 (21%)
2–4 days	19 (25%)
5–7 days	40 (53%)
2 weeks	1 (1%)
**5. How frequently do you recharge your charger?**	
Every day	6 (8%)
Every week	32 (42%)
Every 2 weeks	18 (24%)
Every 4 weeks	12 (15%)
Not fixed	8 (11%)

**Table 4:** Recharging process for patients with rechargeable implanted pulse generators (r-IPGs) (*N* = 76) (Question 6–9).

**6. At what level of battery capacity do you usually recharge your r-IPG?**	
75–100%	28 (37%)
75–50%	38 (50%)
<50%	10 (13%)
Warning sign	0
**7. How long does recharging usually take?**	
<15 min	6 (8%)
15–30 min	15 (20%)
30–45 min	9 (12%)
45–60 min	8 (10%)
More than 60 min	38 (50%)
**8. Do you check and recharge your r-IPG yourself?**	
No	14 (18%)
Yes	62 (82%)
**9. Have you ever been unable to recharge your battery?**	
No	66 (87%)
Yes	10 (13%)
9.1. if yes, could you solve the problem on your own?	
No	5 (50%)
Yes	5 (50%)

### Life With an r-IPG

During the routine recharging process, most of the r-IPG patients (82%, 62/76) preferred to sit or lie down instead of moving around. Approximately half of the r-IPG patients (43%, 33/76) reported needing to recharge while traveling at least once after implantation, and 79% (26/33) had recharged their battery during a vacation. Of the r-IPG patients, 41% (31/76) continued their professional occupation after the surgery, and 32% (10/31) of them had recharged their IPG at work (Table 5).

**Table 5:** Life with a rechargeable implanted pulse generator (r-IPG) (*N* = 76).

**Questions**	**Number (%)**
**1. Have you traveled since your DBS surgery?**	
No	43 (57%)
Yes	33 (43%)
** 1.1. If yes, have you ever recharged during a trip?**	
No	7 (21%)
Yes	26 (79%)
**2. Do you continue to work since DBS surgery?**	
No	45 (59%)
Yes	31 (41%)
**2.1. If yes, have you ever recharged during work?**	
No	21 (68%)
Yes	10 (32%)
**3. Are you ambulatory during recharging?**	
No	62 (82%)
Yes	14 (18%)

*DBS, deep brain stimulation*.

### IPG Replacement

Out of 24 patients who chose the nr-IPG, 20 patients underwent 33 battery replacement procedures, for a mean number of battery replacements per patient of 1.15 times. None of the 100 patients underwent a change of the IPG type (from r-IPG to nr-IPG or vice versa).

## Discussion

In this report, we present the patient preference and satisfaction with different kinds of IPGs (r-IPG and nr-IPG) for the treatment of hyperkinetic movement disorders. All 100 patients reported considering economic factors first when choosing between an r-IPG or nr-IPG, and they noted that an r-IPG was preferred if it was affordable. The rate of satisfaction with the clinical effect of the device did not differ significantly between patients with r-IPGs and nr-IPGs. In patients with r-IPGs, the majority checked the battery capacity every week and recharged every 5–7 days; the recharge usually took more than 60 min. Understanding the recharge process was not difficult for most patients, but some required a long time to feel comfortable with it. Regarding work and travel, r-IPGs did not have a significant adverse impact on patients' daily lives.

The median follow-up in the r-IPG group was 13 months whereas it was 30 months in the nr-IPG group. The reason for the significantly shorter median follow-up of r-IPGs is that nr-IPGs had been adopted and widely used in China notably earlier than r-IPGs. The nr-IPG was first introduced to China in 1999, while the r-IPG was first introduced in 2014 (17). Furthermore, in the initial years after its introduction, the r-IPG was prohibitively expensive for Chinese patients with an average income. Therefore, at our center, it was not until 2016 that patients started to choose the r-IPG, leading to the shorter follow-up period.

In China, the price of the nr-IPG varies from 80,000 RMB to 130,000 RMB, while that of the r-IPG varies from 200,000 RMB to 210,000 RMB. In Shanghai, where our center is located, Chinese national basic medical insurance can cover 50,000 RMB of each IPG's cost, regardless of the type. Therefore, the cost of the nr-IPG ranges from 30,000 RMB to 80,000 RMB, while that of the r-IPG ranges from 150,000 RMB to 160,000 RMB. In consideration of the Chinese average annual income (10410 USD in 2019), the r-IPG would be prohibitively expensive for the majority of the patients. Accordingly, all patients initially consider these economic factors when choosing IPG.

The majority of patients (87/100) were satisfied with their in-service-IPG, and the satisfaction rate did not differ significantly between patients with r-IPGs and nr-IPGs, which indicates that the clinical effect depends on the surgery process rather than on the type of IPG. Among the unsatisfied patients (13/100), an unsatisfactory clinical effect accounted for 69.2%. Among the remaining four patients, two with r-IPGs complained about the inconvenience of programming and the lack of ability to recharge independently, and two patients with nr-IPGs complained about the large device size and the inconvenience of battery-replacement surgery. For patients who could not afford an r-IPG, the nr-IPG meant a lower cost but also less convenience with regard to the inevitable need for replacement.

The majority of the patients with r-IPGs could check and recharge the battery themselves. Of these patients with hyperkinetic movement disorders, 82% were capable of checking and recharging the battery independently, which is higher than the 71.4% reported in patients with PD. The higher proportion may be related to the cognitive problems associated with PD and indicates that patients with hyperkinetic movement disorders are more independent in their daily lives, allowing them to handle the recharging of their devices. As 89% of patients reported feeling confident using their devices, which is similar to the proportion of patients who reported the same with PD (92.7%), the recharging process seems simple. However, it took a long time for some patients to learn (36% took more than 4 weeks), indicating the importance of education surrounding the recharge process. Most patients checked the battery once a week, which is reasonable, while 17% of patients with an r-IPG checked the battery every 2 weeks or longer (7% every 2 weeks, 5% every 4 weeks, and 5% every year). As 99% of patients reported recharging the battery at least every week, we can assume that the 17% mentioned above recharged their IPG without checking the battery. This indicates that it may be unnecessary to check the battery status if a patient recharges the battery regularly. However, checking the status of the r-IPG may help avoid IPG depletion, which would require more than 5 h of continuous charge to restore the power. IPG depletion may also lead to a DBS off-period, which, if not discovered soon enough, may lead to symptom recurrence or exacerbation. In this survey, 13% of the patients were unable to recharge the battery at some point. Half of the participants could recharge the IPG themselves, but the other half required help from a caregiver. If these patients do not receive the needed help in time, the above-mentioned consequences may occur. At our center, we recommend that patients recharge the battery before the capacity falls below 50%. According to this survey, 87% of the patients complied with our advice. In conclusion, to avoid IPG depletion, we recommend that patients pay equal attention to checking and recharging the battery.

Patients may assume that an r-IPG will disrupt their daily life; however, according to our survey, 43% of the patients traveled with an r-IPG, and 41% of patients continued their professional occupations; 32% were able to work and recharge their battery at the same time. Therefore, although an r-IPG may cause inconvenience in patients to a certain extent, the inconvenience is tolerable in most situations.

We are not the first center to conduct this survey; there have been previous studies focusing on patient preference and satisfaction with r-IPGs. Hitti et al. surveyed 206 patients (70% with PD, 20% with essential tremor, and 6% with dystonia) and found that r-IPGs cost less than nr-IPGs when taking into consideration the cost of replacement (13). They reported that 87.3% of the patients were satisfied with an r-IPG, while 6.7% showed difficulty in using the recharge device. This is similar to our findings; however, the present study is the first to focus on hyperkinetic movement disorders.

Furlanetti et al. reported that the main reasons for choosing the nr-IPG were convenience and concern about forgetting to recharge; however, patients using r-IPG did not report experiencing this problem (18). In our study, the main reason for choosing an nr-IPG was the economic cost (92% of patients with nr-IPGs and 62% of patients with r-IPGs). Only ~50% of patients (52% for r-IPG and 54% for nr-IPG) expressed concern over the recharge process when choosing their IPG. It is evident that r-IPGs are less affordable in China, which prohibits many patients from considering the experience and aesthetics when making their choice.

Regarding understanding the recharge process, Jakobs et al. reported that, among 31 patients with movement disorders (21 with PD, 8 with an essential tremor, and 2 with dystonia), 90.3% felt confident using their IPG after a mean of 2.1 weeks and 1.6 training sessions (16). In our study, 57% of patients with hyperkinetic movement disorders felt confident using their IPG within 2 weeks, and 36% required more than 4 weeks. At our center, it is common to offer only one training session on r-IPGs. In light of our findings, we believe that increasing the number of training sessions to 2 or 3 is necessary and beneficial for the patients and their caregivers.

Our research may provide helpful advice regarding the process of introducing the different types of IPGs to patients and may help clinicians to choose an IPG that is more suitable for a patient. We also highlight the importance of the training sessions, and we think that it is necessary to increase the number of training sessions until patients fully understand the use of the recharge device. Moreover, our research could guide the product improvement of the company to produce more convenient and cheaper IPGs and recharge devices.

### Limitations

There are several limitations to our study. First, the male to female ratio differs between the r-IPG and nr-IPG groups. This may have been a result of the small sample size and the sampling bias, and we believe that sex-based differences need to be further analyzed. Consideration of economic factors is inevitable for patients when making the choice between r-IPG and nr-IPG. As mentioned earlier, the cost of the r-IPG is more than twice that of the nr-IPG. Therefore, we believe that the patients' socioeconomic background could be a significant confounder. The proportion of the total cost of DBS treatment borne by the national healthcare insurance system may differ according to country; thus, the importance of economic factors may also vary. Hence, the result of our study may only be applicable to the situation in China. Finally, in this study, we analyzed only the patients' attitude toward the different types of IPG and the recharge phase, without a comprehensive clinical assessment (rate of improvement of symptoms and the stimulation parameters). Given the importance of clinical improvement to the patients' satisfaction, we believe that further studies are needed.

### Conclusion

In this study, we investigated the preference and satisfaction rate for r-IPGs and nr-IPGs among patients with hyperkinetic movement disorders. The results indicate that the majority of the patients were satisfied with their in-service IPG, regardless of whether it was an r-IPG or nr-IPG. The recharge process remains a challenge for some patients and increasing the number of training sessions for the device may be helpful.

## Data Availability Statement

The raw data supporting the conclusions of this article will be made available by the authors, without undue reservation.

## Ethics Statement

Ethical review and approval was not required for the study on human participants in accordance with the local legislation and institutional requirements. Written informed consent for participation was not required for this study in accordance with the national legislation and the institutional requirements.

## Author Contributions

XQ: conception, organization, execution of research project, and writing of the first draft of manuscript. YuhW: organization, execution of research project, review and critique of statistical analysis, and writing of the first draft of manuscript. ZL: execution of research project, design, execution of statistical analysis, and writing of the first draft of manuscript. YunW and WX: organization and execution of research project. YiW and BS: conception of research project and review and critique of manuscript. KA: conception of research project. CZ and DL: conception, organization of research project, and review and critique of manuscript. All authors contributed to the article and approved the submitted version.

## Conflict of Interest

The authors declare that the research was conducted in the absence of any commercial or financial relationships that could be construed as a potential conflict of interest.
