# Corrigendum: Fixed-life or rechargeable batteries for deep brain stimulation: preference and satisfaction among patients with hyperkinetic movement disorders

**DOI:** 10.3389/fneur.2023.1309569

**Published:** 2023-12-29

**Authors:** Xian Qiu, Yuhan Wang, Zhengyu Lin, Yunhao Wu, Wenying Xu, Yiwen Wu, Bomin Sun, Keyoumars Ashkan, Chencheng Zhang, Dianyou Li

**Affiliations:** ^1^Department of Nursing, Ruijin Hospital, Shanghai Jiao Tong University School of Medicine, Shanghai, China; ^2^Department of Neurosurgery, Ruijin Hospital, Shanghai Jiao Tong University School of Medicine, Shanghai, China; ^3^Department of Neurology, Ruijin Hospital, Shanghai Jiao Tong University School of Medicine, Shanghai, China; ^4^Department of Neurosurgery, King's College Hospital, London, United Kingdom

**Keywords:** movement disorders, implantable pulse generators, deep brain stimulation, hyperkinetic movement disorders, dystonia, Tourette syndrome

In the published article “Jakobs M, Kloß M, Unterberg A, Kiening K. Rechargeable internal pulse generators as initial neurostimulators for deep brain stimulation in patients with movement disorders. *Neuromodulation*. (2018) 21:604–10” was not cited in the article. The citation has now been inserted in **Materials and Methods**, **Questionnaire**, and should read:

“An Internet-based questionnaire (powered by www.wjx.cn) was developed and distributed via the online chat program, WeChat. The questionnaire was designed with reference to the research of Jakobs et al. (16), and were adjusted according to the situation of Chinese patients. The questions covered patient demographics, factors that impacted the patient's choice, the patient's satisfaction with their choice, and DBS surgery. In particular, several questions were designed specifically for patients implanted with an r-IPG device; they inquired about the feasibility and reliability of the battery recharge, the interval between recharges, the duration of the recharge process, and the convenience of postoperative r-IPG management. The questionnaire was distributed via the online chat platform WeChat. Participants completed the questionnaire after having received at least 8 months of DBS treatment. In most cases, it took no more than 30 min to complete the questionnaire.”

The authors apologize for this error and state that this does not change the scientific conclusions of the article in any way. The original article has been updated.

