# Supplementary material for: Fixed-Life or Rechargeable Batteries for Deep Brain Stimulation: Preference and Satisfaction Among Patients With Hyperkinetic Movement Disorders
Source: Front Neurol. 2021 May 28;12:662383. doi: 10.3389/fneur.2021.662383 (PMC8193684; doi:10.3389/fneur.2021.662383)
Supplement: Supplementary file 1 [file Table_1.docx]

**Supplementary Table 1. The questionnaire ‘Preference and Satisfaction of fixed-life and rechargeable batteries of DBS among patients with hyperkinetic movment disorders’**

**General Information**

| **Questions** |
| --- |
|  |
|  |
| **1. Name:_____________; 2. Birthday: ______________; 3.** **Contact:_____________;** |
| **4. Gender: ____________; 5.Education years: _______________;** |
| **6.Diagnosis:** |
| Dystonia |
| Tourette syndrome |
| **7. When you have your last IPG implanted?** |
| **8. How much is your budget for DBS before the surgery?** |
| **9. What is your type of IPG, rechargeable or non-rechargeable?** |
| Rechargeable. |
| Non-rechargeable. |
| **10. Which brand of the IPG are you using now?** |
| **11. Which are the main reasons choosing the IPG you have been using?** |
| Economic factor |
| Brand |
| Tele-programming |
| Doctors’ recommendation |
| Other patients’ recommendation |
| Advertisement |
| Inconvenience of recharge |
| Inconvenience of replacement of IPG |
| other |
| **12. Regardless of other factors, if you use the r-IPG, how often would you prefer (convenient for you) to have the IPG recharged?** |
| Every day |
| Every week |
| Every two weeks |
| Every month |
| Every year |
| **13. Regardless of other factors, if you use the r-IPG, how long could you accept (convenient for you) for every IPG recharge?** |
| Less than 15 min |
| 15–30 min |
| 30–45 min |
| 45–60 min |
| More than 60 min |

**Satisfaction rate**

| **Questions** |
| --- |
|  |
|  |
| **1. Are you still happy with your choice of device?** |
| Yes |
| No |
| **1.1. If not, please specify the reason.** |
| The stimulating effects did not meet your expectations. |
| Other |
| **2. Would you choose the same type of device today?** |
| Yes |
| No |

**Recharging process for patients with rechargeable implanted pulse generators**

| **Questions** |
| --- |
| **1. Do you feel confident using your r-IPG?** |
| No |
| Yes |
| **1.1. If yes, how long did it take for you to feel confident?** |
| Less than 1 week |
| 1–2 weeks |
| 2–4 weeks |
| More than 4 weeks |
| **2. How frequently do you check the battery capacity of your r-IPG?** |
| Every day |
| Every week |
| Every 2 weeks |
| Every 4 weeks |
| Every year |
| **3. Do you ever forget to recharge your r-IPG?** |
| No |
| Yes |
| **4. How frequently do you recharge your r-IPG?** |
| Every day |
| 2–4 days |
| 5–7 days |
| 2 weeks |
| **5. How frequently do you recharge your charger?** |
| Every day |
| Every week |
| Every 2 weeks |
| Every 4 weeks |
| Not fixed |
| **6. At what level of battery capacity do you usually recharge your r-IPG?** |
| 75–100% |
| 75–50% |
| < 50% |
| Warning sign |
| **7. How long does recharging usually take?** |
| Less than 15 min |
| 15–30 min |
| 30–45 min |
| 45–60 min |
| More than 60 min |
| **8. Do you check and recharge your r-IPG yourself?** |
| No |
| Yes |
| **9. Have you ever been unable to recharge your battery?** |
| No |
| Yes |
| **9.1. if yes, could you solve the problem on your own?** |
| No |
| Yes |

**Life with a rechargeable implanted pulse generator (r-IPG)**

| **Questions** |
| --- |
| **1. Have you traveled since your DBS surgery?** |
| No |
| Yes |
| **1.1. If yes, have you ever recharged during a trip?** |
| No |
| Yes |
| **2. Do you continue to work since DBS surgery?** |
| No |
| Yes |
| **2.1. If yes, have you ever recharged during work?** |
| No |
| Yes |
| **3. Are you ambulatory during recharging?** |
| No |
| Yes |
