# Supplementary material for: Fixed-Life or Rechargeable Batteries for Deep Brain Stimulation: Preference and Satisfaction Among Patients With Hyperkinetic Movement Disorders
Source: Front Neurol. 2021 May 28;12:662383. doi: 10.3389/fneur.2021.662383 (PMC8193684; doi:10.3389/fneur.2021.662383)
Supplement: Supplementary file 2 [file Table_2.docx]

**Supplementary Table 2. The analysis of the result of patients with dystonia**

**Supplementary Table 2-1. Satisfaction rate of DBS in patients with dystonia**

| **Questions** | **Group** | |
| --- | --- | --- |
|  | **r-IPG** | **nr-IPG** |
|  | **(N = 59)** | **(N = 20)** |
| **1. Are you still happy with your choice of device?** |  |  |
| Yes | 53 (89.8%) | 19 (95%) |
| No | 6 (10.2%) | 1 (5%) |
| **1.1. If not, please specify the reason.** |  |  |
| The stimulating effects did not meet your expectations. | 5 (8.5%) | 0 (0%) |
| Other | 1 (1.7%) | 1 (25%) |
| **2. Would you choose the same type of device today?** |  |  |
| Yes | 50 (84.7%) | 19 (95%) |
| No | 9 (15.3%) | 1 (5%) |

r-IPG: rechargeable implanted pulse generator; nr-IPG: non-rechargeable implanted pulse generator.

**Supplementary Table 2-2. Recharging process for patients suffered from dystonia with rechargeable implanted pulse generators (r-IPGs) (N = 59)**

| **Questions** | **Number (%)** |
| --- | --- |
| **1. Do you feel confident using your r-IPG?** |  |
| No | 5 (8.5%) |
| Yes | 54 (91.5%) |
| **1.1. If yes, how long did it take for you to feel confident?** |  |
| Less than 1 week | 21 (35.6%) |
| 1–2 weeks | 10 (16.9%) |
| 2–4 weeks | 5 (8.5%) |
| More than 4 weeks | 18 (30.5%) |
| **2. How frequently do you check the battery capacity of your r-IPG?** |  |
| Every day | 8 (13.6%) |
| Every week | 42 (71.2%) |
| Every 2 weeks | 4 (6.8%) |
| Every 4 weeks | 2 (3.4%) |
| Every year | 3 (5.1%) |
| **3. Do you ever forget to recharge your r-IPG?** |  |
| No | 45 (76.3%) |
| Yes | 14 (23.7%) |
| **4. How frequently do you recharge your r-IPG?** |  |
| Every day | 11 (18.6%) |
| 2–4 days | 11 (18.6%) |
| 5–7 days | 36 (61.0%) |
| 2 weeks | 1 (1.7%) |
| **5. How frequently do you recharge your charger?** |  |
| Every day | 4 (6.8%) |
| Every week | 27 (45.8%) |
| Every 2 weeks | 14 (23.7%) |
| Every 4 weeks | 10 (16.9%) |
| Not fixed | 4 (6.8%) |
| **6. At what level of battery capacity do you usually recharge your r-IPG?** |  |
| 75–100% | 21 (35.6%) |
| 75–50% | 31 (52.5%) |
| < 50% | 7 (11.9%) |
| Warning sign | 0 |
| **7. How long does recharging usually take?** |  |
| Less than 15 min | 5 (8.5%) |
| 15–30 min | 9 (15.3%) |
| 30–45 min | 6 (10.2%) |
| 45–60 min | 7 (11.9%) |
| More than 60 min | 32 (54.2%) |
| **8. Do you check and recharge your r-IPG yourself?** |  |
| No | 10 (16.9%) |
| Yes | 49 (83.1%) |
| **9. Have you ever been unable to recharge your battery?** |  |
| No | 54 (91.5%) |
| Yes | 5 (8.5%) |
| **9.1. if yes, could you solve the problem on your own?** |  |
| No | 2 (40%) |
| Yes | 3 (60%) |

**Supplementary Table 2-3. Life with a rechargeable implanted pulse generator in patients with dystonia (r-IPG) (N = 59)**

| **Questions** | **Number (%)** |
| --- | --- |
| **1. Have you traveled since your DBS surgery?** |  |
| No | 34 (57.6%) |
| Yes | 25 (42.4%) |
| **1.1. If yes, have you ever recharged during a trip?** |  |
| No | 6 (12.5%) |
| Yes | 19 (87.5%) |
| **2. Do you continue to work since DBS surgery?** |  |
| No | 34 (57.6%) |
| Yes | 25 (42.4%) |
| **2.1. If yes, have you ever recharged during work?** |  |
| No | 19 (76.0%) |
| Yes | 6 (24.0%) |
| **3. Are you ambulatory during recharging?** |  |
| No | 51 (86.4%) |
| Yes | 8 (13.6%) |

DBS: deep brain stimulation
