# Supplementary material for: Fixed-Life or Rechargeable Batteries for Deep Brain Stimulation: Preference and Satisfaction Among Patients With Hyperkinetic Movement Disorders
Source: Front Neurol. 2021 May 28;12:662383. doi: 10.3389/fneur.2021.662383 (PMC8193684; doi:10.3389/fneur.2021.662383)
Supplement: Supplementary file 3 [file Table_3.docx]

**Supplementary Table 3. The analysis of the result of patients with Tourette syndrome**

**Supplementary Table 3-1. Satisfaction rate of DBS in patients with Tourette Syndrome**

| **Questions** | **Group** | |
| --- | --- | --- |
|  | **r-IPG** | **nr-IPG** |
|  | **(N = 17)** | **(N = 4)** |
| **1. Are you still happy with your choice of device?** |  |  |
| Yes | 12 (71%) | 3 (75%) |
| No | 5 (29%) | 1 (25%) |
| **1.1. If not, please specify the reason.** |  |  |
| The stimulating effects did not meet your expectations. | 4 (29%) | 0 |
| Other | 1 (6%) | 1 (25%) |
| **2. Would you choose the same type of device today?** |  |  |
| Yes | 12 (71%) | 3 (75%) |
| No | 5 (29%) | 1 (25%) |

r-IPG: rechargeable implanted pulse generator; nr-IPG: non-rechargeable implanted pulse generator.

**Supplementary Table 3-2. Recharging process for patients suffered from Tourette syndrome with rechargeable implanted pulse generators (r-IPGs) (N = 17)**

| **Questions** | **Number (%)** |
| --- | --- |
| **1. Do you feel confident using your r-IPG?** |  |
| No | 7 (41.2%) |
| Yes | 10 (58.8%) |
| **1.1. If yes, how long did it take for you to feel confident?** |  |
| Less than 1 week | 5 (29.4%) |
| 1–2 weeks | 3 (17.6%) |
| 2–4 weeks | 6 (35.3%) |
| More than 4 weeks | 3 (17.6%) |
| **2. How frequently do you check the battery capacity of your r-IPG?** |  |
| Every day | 5 (29.4%) |
| Every week | 8 (47.1%) |
| Every 2 weeks | 1 (5.9%) |
| Every 4 weeks | 2 (11.8%) |
| Every year | 1 (5.9%) |
| **3. Do you ever forget to recharge your r-IPG?** |  |
| No | 10 (58.8%) |
| Yes | 7 (41.2%) |
| **4. How frequently do you recharge your r-IPG?** |  |
| Every day | 5 (29.4%) |
| 2–4 days | 8 (47.1%) |
| 5–7 days | 4 (23.5%) |
| 2 weeks | 0 (0%) |
| **5. How frequently do you recharge your charger?** |  |
| Every day | 2 (11.8%) |
| Every week | 5 (29.4%) |
| Every 2 weeks | 4 (23.5%) |
| Every 4 weeks | 2 (11.8%) |
| Not fixed | 4 (23.5%) |
| **6. At what level of battery capacity do you usually recharge your r-IPG?** |  |
| 75–100% | 7 (41.2%) |
| 75–50% | 7 (41.2%) |
| < 50% | 3 (17.6%) |
| Warning sign | 0 |
| **7. How long does recharging usually take?** |  |
| Less than 15 min | 1 (5.9%) |
| 15–30 min | 6 (35.3%) |
| 30–45 min | 3 (17.6%) |
| 45–60 min | 1 (5.9%) |
| More than 60 min | 6 (35.5%) |
| **8. Do you check and recharge your r-IPG yourself?** |  |
| No | 4 (23.5%) |
| Yes | 13 (76.5%) |
| **9. Have you ever been unable to recharge your battery?** |  |
| No | 12 (70.6%) |
| Yes | 5 (29.4%) |
| **9.1. if yes, could you solve the problem on your own?** |  |
| No | 3 (50%) |
| Yes | 2 (50%) |

**Supplementary Table 3-3. Life with a rechargeable implanted pulse generator in patients with Tourette syndrome (r-IPG) (N = 17)**

| **Questions** | **Number (%)** |
| --- | --- |
| **1. Have you traveled since your DBS surgery?** |  |
| No | 9 (52.9%) |
| Yes | 8 (47.1%) |
| **1.1. If yes, have you ever recharged during a trip?** |  |
| No | 1 (12.5%) |
| Yes | 7 (87.5%) |
| **2. Do you continue to work since DBS surgery?** |  |
| No | 11 (64.7%) |
| Yes | 6 (35.3%) |
| **2.1. If yes, have you ever recharged during work?** |  |
| No | 2 (33.3%) |
| Yes | 4 (66.7%) |
| **3. Are you ambulatory during recharging?** |  |
| No | 12 (70.6%) |
| Yes | 4 (29.4%) |

DBS: deep brain stimulation
